# Supplementary material for: A Diet Induced Maladaptive Increase in Hepatic Mitochondrial DNA Precedes OXPHOS Defects and May Contribute to Non-Alcoholic Fatty Liver Disease
Source: Cells. 2019 Oct 8;8(10):1222. doi: 10.3390/cells8101222 (PMC6830072; doi:10.3390/cells8101222)
Supplement: Supplementary file 1 [file cells-08-01222-s001.pdf]

cells

Supplementary information—Analysis of proteins by mass spectrometry

Quantitative mass spectrometry analysis.

Lysis buffer (8 M Urea, 1% SDS, 50 mM Tris pH 8.5, Protease and Phosphatase inhibitors) was added to the liver samples to achieve a cell lysate with a protein concentration between 2-8 mg/mL. Proteins were reduced and alkylated as previously described. Proteins were precipitated using methanol/chloroform. In brief, four volumes of methanol were added to the cell lysate, followed by one volume of chloroform, and finally three volumes of water. The mixture was vortexed and centrifuged to separate the chloroform phase from the aqueous phase. The precipitated protein was washed with one volume of ice-cold methanol. The washed precipitated protein was allowed to air dry. Precipitated protein was resuspended in 4 M Urea, 50 mM Tris pH 8.5. Proteins were first digested with LysC (1:50; enzyme:protein) for 12 hours at 25 oC. The LysC digestion is diluted down to 1 M Urea, 50 mM Tris pH8.5 and then digested with trypsin (1:100; enzyme:protein) for another 8 hours at 25 oC. Peptides were desalted using a C18 solid phase extraction cartridges as previously described. Dried peptides were resuspended in 200 mM EPPS, pH 8.0. Peptide quantification was performed using the micro-BCA assay (Pierce). The same amount of peptide from each condition was labeled with tandem mass tag (TMT) reagent (1:4; peptide: TMT label, Pierce). The 6-plex (brain tissue) and 10-plex (other tissues) labeling reactions were performed for 2 hours at 25 oC. Modification of tyrosine residue with TMT was reversed by the addition of 5% hydroxylamine for 15 minutes at 25 oC. The reaction was quenched with 0.5% TFA and samples were combined at a 1:1:1:1:1:1:1:1:1:1 ratio for 10-plex experiments. Combined samples were desalted and fractionated offline into 24 fractions as previously described (Weekes et al., 2014).

Liquid chromatography-MS3 spectrometry (LC-MS/MS)

12 of the 24 peptide fractions from the basic reverse phase step (every other fraction) were analyzed with an LC-MS3 data collection strategy (McAlister et al., 2014) on an Orbitrap Fusion mass spectrometer (Thermo Fisher Scientific Inc., Waltham, MA, USA) equipped with a Proxeon Easy nLC 1000 for online sample handling and peptide separations. Approximately 5 µg of peptide resuspended in 5% formic acid + 5% acetonitrile was loaded onto a 100 µm inner diameter fused-silica micro capillary with a needle tip pulled to an internal diameter less than 5 µm. The column was packed in-house to a length of 35 cm with a C18 reverse phase, resin (GP118 resin 1.8 µm, 120 Å, Sepax Technologies Inc., Newark, DE, USA). The peptides were separated using a 120 min linear gradient from 3% to 25% buffer B (100% ACN + 0.125% formic acid) equilibrated with buffer A (3% ACN + 0.125% formic acid) at a flow rate of 600 nL/min across the column. The scan sequence for the Fusion Orbitrap began with an MS1 spectrum (Orbitrap analysis, resolution 120,000, 400−1400 m/z scan range, AGC target 2 × 105, maximum injection time 100 ms, dynamic exclusion of 75 seconds). 'Top speed' (2 second) was selected for MS2 analysis, which consisted of CID (quadrupole isolation set at 0.5 Da and ion trap analysis, AGC 4 × 103, NCE 35, maximum injection time 150 ms). The top ten precursors from each MS2 scan were selected for MS3 analysis (synchronous precursor selection), in which precursors were fragmented by HCD prior to Orbitrap analysis (NCE 55, max AGC 5 × 104, maximum injection time 150 ms, isolation window 2.5 Da, resolution 60,000.

LC-MS3 data analysis

A suite of in-house software tools were used for RAW file processing and controlling peptide and protein level false discovery rates, assembling proteins from peptides, and protein quantification from peptides as previously described. MS/MS spectra were searched against a Uniprot mouse database (2014) with both the forward and reverse sequences. Database search criteria are as follows: tryptic with two missed cleavages, a precursor mass tolerance of 50 ppm, fragment ion mass tolerance

of 1.0 Da, static alkylation of cysteine (57.02146 Da), static TMT labelling of lysine residues and N-termini of peptides (229.162932 Da), and variable oxidation of methionine (15.99491 Da). TMT reporter ion intensities were measured using a 0.003 Da window around the theoretical m/z for each reporter ion in the MS3 scan for 10-plex data (0.03 for 6-plex data). Peptide spectral matches with poor quality MS3 spectra were excluded from quantitation (<200 summed signal-to-noise across 10 channels and <0.5 precursor isolation specificity).

Weekes, M.P., Tomasec, P., Huttlin, E.L., Fielding, C.A., Nusinow, D., Stanton, R.J., Wang, E.C., Aicheler, R., Murrell, I., Wilkinson, G.W., Lehner, P.J., Gygi, S.P. (2014) Quantitative temporal viromics: an approach to investigate host-pathogen interaction. Cell 157:1460-1472.

McAlister, G.C., Nusinow, D.P., Jedrychowski, M.P., Wühr, M., Huttlin, E.L., Erickson, B.K., Rad, R., Haas, W., Gygi, S.P. (2014) MultiNotch MS3 enables accurate, sensitive, and multiplexed detection of differential expression across cancer cell line proteomes. Anal. Chem. 86:7150-8.

Table S1: Composition of Standard and High-fat diets

	Standard diet (%)	High-fat diet (%)
Protein	19%	20.7%
Fibre	4.9%	5%
Ash	6.4%	5.6%
N free extracts	54.1%	34.3%
Starch	36.5%	17.2%
Sugar	4.7%	16.3%
Fat	3.3%	30%
saturated	0.6%	14.2%
monounsaturated	0.7%	11.7%
polyunsaturated	2.3%	0.95%

According to the table, diets have distinct compositions mostly in terms of Nitrogen- free extracts, starch, sugar and fat. Relatively to the fat content, there is an enrichment of saturated and monounsaturated fatty acids in the High-fat diet in comparison with the control diet. There are no differences between the fat content of the High-fat diet (HFD) and the Western diet (WD). The only difference between HFD and WD is that the WD was enriched in 30% of sugar in the drinking water.

Table S2: Diet-induced alterations in protein levels in steatotic mouse liver

		Chow	High Fat		Western diet	
		Mean (SD)	Mean (SD)	Fold change[1]	Mean (SD)	Fold change[1]
mtDNA-encoded OXPHOS proteins						
MTND1	NU1M_MOUSE NADH-ubiquinone oxidoreductase chain 1	7.73 (1.87)	12.18 (0.39)	↑1.57x	10.51 (0.79)	-
MTND2	NU2M_MOUSE NADH-ubiquinone oxidoreductase chain 2	12.94 (1.29)	9.36 (3.06)	-	7.43 (2.36)	↓0.57x
MTND4	NU4M_MOUSE NADH-ubiquinone oxidoreductase chain 4	9.89 (0.24)	10.25 (0.44)	-	9.85 (0.78)	-
MTND5	NU5M_MOUSE NADH-ubiquinone oxidoreductase chain 5	9.73 (1.18)	11.04 (0.72)	-	9.59 (0.96)	-
MTCO1	COX1_MOUSE Cytochrome c oxidase subunit 1	11.21 (1.38)	10.73 (1.81)	-	8.10 (1.56)	-
MTCO2	COX2_MOUSE Cytochrome c oxidase subunit 2	10.23 (0.31)	10.21 (0.34)	-	9.34 (0.72)	-
MTCO3	COX3_MOUSE Cytochrome c oxidase subunit 3	11.87 (2.31)	10.37 (1.63)	-	7.30 (1.58)	↓0.61x
MTCYB	CYB_MOUSE Cytochrome b	8.94 (2.34)	11.52 (0.33)	-	9.97 (0.82)	-
MTATP6	ATP6_MOUSE ATP synthase subunit a	8.70 (2.27)	11.89 (0.15)	-	9.52 (4.83)	-
MTATP8	ATP8_MOUSE ATP synthase protein 8	9.83 (1.20)	11.21 (0.70)	-	9.21 (2.07)	-
Nuclear DNA-encoded OXPHOS proteins						
NDUFB8	NDUB8_MOUSE NADH dehydrogenase [ubiquinone] 1 beta subcomplex subunit 8, mitochondrial	9.65 (0.36)	10.70 (0.43)	↑1.11x	9.66 (0.59)	-
SDHA	SDHA_MOUSE Succinate dehydrogenase [ubiquinone] flavoprotein subunit, mitochondrial	11.02 (0.32)	9.96 (0.31)	↓0.90x	9.12 (0.63)	↓0.83x
UQCRB	QCR7_MOUSE Cytochrome b-c1 complex subunit 7	10.57 (0.34)	10.42 (0.22)	-	9.18 (0.11)	↓0.87x
COX5B	COX5B_MOUSE Cytochrome c oxidase subunit 5B, mitochondrial	9.27 (0.62)	11.25 (0.40)	↑1.21x	9.70 (0.22)	-
ATP5A1	ATPA_MOUSE ATP synthase subunit alpha, mitochondrial	10.44 (0.34)	10.25 (0.37)	-	9.39 (0.39)	↓0.90x
mtDNA replication machinery						
POLG2	DPOG2_MOUSE DNA polymerase subunit gamma-2, mitochondrial	13.12 (0.60)	10.31 (1.57)	↓0.79x	6.81 (1.48)	↓0.52x
HELB	HELB_MOUSE DNA helicase B	9.70 (0.87)	10.53 (0.69)	-	9.67 (0.91)	-
MTPAP	PAPD1_MOUSE Poly(A) RNA polymerase, mitochondrial	12.06 (1.26)	8.82 (0.62)	↓0.73	8.81 (0.58)	↓0.73x
SSBP1	SSBP_MOUSE Single-stranded DNA-binding protein, mitochondrial	10.42 (0.52)	10.69 (0.13)	-	8.82 (0.78)	↓0.85x

mtDNA transcription						
TFAM	TFAM_MOUSE Transcription factor A, mitochondrial	9.12 (0.42)	11.00 (0.33)	↑1.21x	9.36 (1.48)	-
Mitochondrial dynamics						
MFN1	MFN1_MOUSE Mitofusin-1	10.99 (0.26)	10.67 (0.71)	-	9.04 (0.45)	↓0.82x
MFN2	MFN2_MOUSE Mitofusin-2	9.57 (0.70)	10.49 (0.38)	-	10.26 (1.19)	-
OPA1	OPA1_MOUSE Isoform 2 of Dynamin-like 120 kDa protein, mitochondrial	10.33 (0.31)	10.39 (0.11)	-	9.21 (0.69)	-
FIS1	FIS1_MOUSE Mitochondrial fission 1 protein	11.60 (1.91)	9.87 (1.15)	-	9.13 (0.54)	-
Methylation						
DNMT1	DNMT1_MOUSE DNA (cytosine-5)-methyltransferase 1	8.87 (0.50)	9.54 (0.45)	-	11.73 (1.03)	↑1.32x
Inflammation						
NFKB1	NFKB1_MOUSE Nuclear factor NF-kappa-B p105 subunit	8.80 (0.29)	9.18 (0.68)	-	12.16 (1.56)	↑1.38x
NFKB2	NFKB2_MOUSE Nuclear factor NF-kappa-B p100 subunit	7.75 (0.50)	9.27 (0.41)	↑1.20x	13.39 (1.12)	↑1.73x
MYD88	MYD88_MOUSE Myeloid differentiation primary response protein MyD88	9.88 (0.28)	10.07 (0.58)	-	9.94 (0.10)	-

[1]Fold change relative to chow levels

SD = standard deviation. N=3 per group.
